# Auditory Sensory Substitution is Intuitive and Automatic with Texture Stimuli

**DOI:** 10.1038/srep15628

**Published:** 2015-10-22

**Authors:** Noelle R. B. Stiles, Shinsuke Shimojo

**Affiliations:** 1 Biology and Biological Engineering, MC 139-74, California Institute of Technology, Pasadena, CA.

## Abstract

Millions of people are blind worldwide. Sensory substitution (SS) devices (*e.g.*, vOICe) can assist the blind by encoding a video stream into a sound pattern, recruiting visual brain areas for auditory analysis via crossmodal interactions and plasticity. SS devices often require extensive training to attain limited functionality. In contrast to conventional attention-intensive SS training that starts with visual primitives (*e.g.*, geometrical shapes), we argue that sensory substitution can be engaged efficiently by using stimuli (such as textures) associated with intrinsic crossmodal mappings. Crossmodal mappings link images with sounds and tactile patterns. We show that intuitive SS sounds can be matched to the correct images by naive sighted participants just as well as by intensively-trained participants. This result indicates that existing crossmodal interactions and amodal sensory cortical processing may be as important in the interpretation of patterns by SS as crossmodal plasticity (*e.g.*, the strengthening of existing connections or the formation of new ones), especially at the earlier stages of SS usage. An SS training procedure based on crossmodal mappings could both considerably improve participant performance and shorten training times, thereby enabling SS devices to significantly expand blind capabilities.

Sensory substitution is used to aid the sensory-impaired such as the blind by encoding the information inaccessible to the lost sense into stimuli of another modality^1^. Devices such as these have translated images into either sound or tactile sensations. The vOICe device is an auditory sensory substitution device that translates vertical position to frequency, left-right position to scan time (encoded in stereo), and brightness to sound loudness^1^ (as shown in Fig. 1a).

Both blindfolded sighted and blind participants can recognize and localize natural and artificial objects with sensory substitution, given that participants have training (training has a wide range of durations and can last up to years) and use top-down attention (Note: training on sensory substitution has not yet been fully optimized or systematized, and therefore is quite variable in duration and outcomes)^2^,^3^,^4^,^5^,^6^,^7^,^8^. Whereas visual perception in the sighted is often effortless and automatic, the interpretation of SS has so far been laborious, requiring a top-down cognitive process (particularly at the beginning of training) providing a barrier to widespread usage by a substantial fraction of the blind population. Meanwhile, neural imaging studies (PET and fMRI) on SS have shown the presence of crossmodal interactions or plasticity, but uncertainty remains as to whether SS is driven by a top-down or a bottom-up process^2^,^6^. TMS studies indicate that visual area activation from sensory substitution is causally linked to blind users’ SS task performance^9^,^10^ showing that the SS-based perceptual process could be bottom-up but not necessarily effortless. There is not yet (as far as we can tell) a clear indication (among behavior or imaging studies) that sensory substitution interpretation can be intuitive^11^,^12^,^13^. Pre-existing crossmodal interactions (or crossmodal mappings) may allow sensory substitution to be intuitive, however, their impact on sensory substitution interpretation has not yet been explored in depth. In fact, pre-existing crossmodal interactions may be as important to SS interpretation (particularly at the beginning of training), as crossmodal plasticity and learning.

Crossmodal correspondence literature has shown that intrinsic mappings exist between visual and auditory stimuli^14^. If intrinsic mappings are included in the sensory substitution device encoding, they may allow participants to perform sensory substitution tasks without any training, effort, or knowledge of the device encoding. The encoding of vOICe is based on long-evidenced correspondences across vision and audition, such as the matching of brightness and loudness intensity^15^, spatial height and pitch height^16^ and, spatial frequency and amplitude-modulation (AM) rate^17^. In addition, other features of the vOICe encoding are intuitive, for example the scanning of a scene from left to right is similar to reading written English (and the blind read braille left to right as well). Therefore, participants with no knowledge about the vOICe device may in principle be able to use crossmodal correspondences to naively match images with their correct vOICe sounds. Certain stimuli such as textures may have strong intrinsic crossmodal associations and similar structure across modalities (as shown in Fig. 1b), and thus may also be correctly interpreted by naive participants.

For clarity, we will refer to all crossmodal interactions existing before these experiments as pre-existing crossmodal interactions; therefore, pre-existing as used herein does not necessarily mean innate. All crossmodal changes engendered through training in these experiments will be referred to as crossmodal plasticity.

We address two crossmodal mapping problems in this paper: the engineering issue of optimally encoding vision into audition (V → A), and the psychological/neural decoding of SS via crossmodal correspondences (A → V). We began by studying the psychological/neural decoding of SS with the existing vOICe device encoding (Fig. 1), to determine if vOICe can be intuitive. The results then suggested optimal methods for the encoding of vision into audition. In other words, once we know what works in vOICe, we can then accentuate those characteristics to make even more intuitive device encodings and training procedures.

Experiment 1 will show that SS can be intuitive to the entirely naive untrained observers, a key finding of this paper. Experiments 2 through 4 will study in detail why this is true, and Expt. 5 will critically extend this result to the blind. Experiments 2 through 5 all support and fortify the result in Expt. 1. A note to blind readers, this set of experiments test for accuracy and automaticity for interpreting images with vOICe, therefore it is a start to making the vOICe device more intuitive, however it cannot entirely address (as no single study can) the vOICe device’s challenges with cluttered environments, and dynamic attention allocation.

## Results

### Expt. 1: Matching Images and vOICe Sounds

#### Introduction

The role of crossmodal correspondences was tested with sighted participants in a bimodal matching experiment (Fig. 2a). Participants heard one sound and were asked to match it to one of three presented images (three alternative forced-choice paradigm; 3AFC). Naive participants were not told the vOICe encoding scheme, nor that the sounds were from the vOICe. Images were grouped in sets of three or four images, of which three were displayed as choices for any given trial. This image grouping was used so that particular image features and types could be tested separately. Image types ranged from natural to artificial images, and from simple to complex images. Image sets included vertical bar textures of different thicknesses, circular patterns of different element sizes, and images of natural textures (Fig. 3). A total of 24 image sets were tested (Fig. 3).

**Figure 1:**
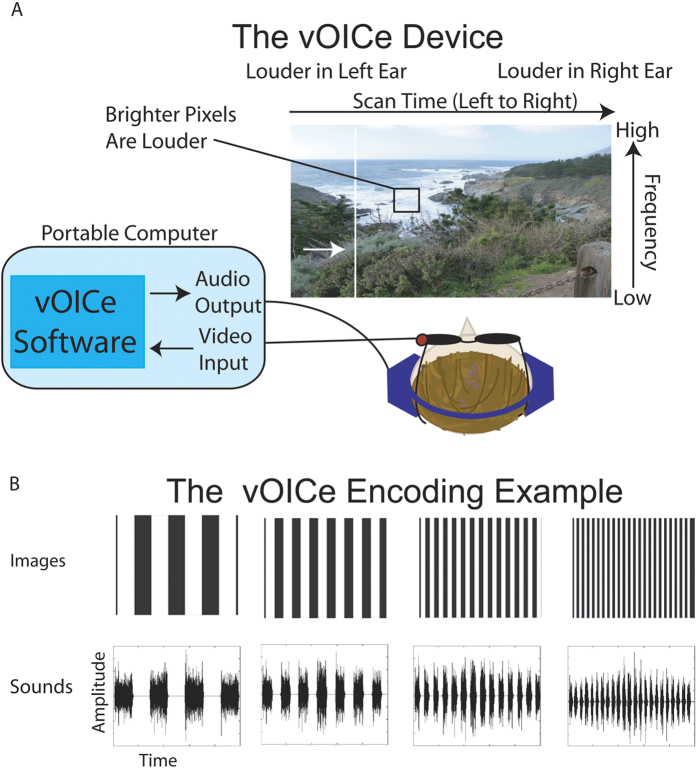
The vOICe device encoding used in the experiments. (**a**) Depicts the vOICe encoding scheme. A camera mounted on glasses records video that is converted to sound by a computer and transmitted to headphones in real time. (**b**) Shows the example output from vOICe for a given set of images used in the experiments. These particular images and vOICe sounds have a similar structure and therefore are intuitive to match. (Further details provided in supplementary methods.). Schematic and Image generated by N. Stiles.

Vertical bar texture images can be matched to the related vOICe sounds by an analytic strategy such as counting the number of elements (similar to the spatial frequency and AM rate correspondence^17^) (Fig. 1b); likewise, the circular patterns (Fig. 3a, second column) could be paired by equating the rate of change of the sound with element size (similar to the Bouba-Kiki effect^18^). Texture discrimination may rely on fine repetitive patterns of sound changes (along both the pitch and the time axes).

#### Results

Naive sighted participants (*N* = 5 to 7 participants, varied across stimulus sets) performed significantly above chance (*p* < 0.002, with Bonferroni multiple comparisons correction) in 16 of 24 images sets tested, trained sighted participants (*N* = 5) in 18 of 24 image sets (all data in Fig. 3), and both the naive and trained participants in 14 of 24 image sets (the naive and trained were not significantly different in any image set). If just non-modified natural stimuli are counted, 5 of 7 image sets were significantly above chance (*p* < 0.002, with Bonferroni multiple comparisons correction) for the naive sighted and 6 of 7 image sets for the trained sighted. When all the stimuli are pooled, both groups revealed well-above-chance performance (Naive: *p* < 2.09 × 10^−121^; Trained: *p* < 9.15 × 10^−119^), and the between-group difference is not significant (Welch’s t-test, *p* < 0.71).

#### Discussion

The result that select natural stimuli could be intuitive with sensory substitution, with or without training, was unexpected. This is especially true considering that natural images are typically considered cluttered and complex, and thus hard to interpret. Natural stimuli (such as natural textures) have more spatial frequency and brightness variations than the typical simplified lab image (a vertical line, for example). The majority of participants being trained on sensory substitution, as reported in the literature, begin with a simplified lab environment, such as a white isolated object on a black background, and only experience a natural environment with the device after several training sessions. Our study indicates that this approach may not be optimal, due to the implicit assumption that natural stimuli can only be interpreted with training. We have found that some natural stimuli (such as natural textures) are rich in crossmodal correspondences, and therefore are easy to interpret with vOICe. This finding suggests that training participants with a crossmodal-correspondence-rich environment that includes not only simplified lab stimuli/tasks but also natural texture tasks may prove to be a more effective strategy.

Several studies have concluded that crossmodal plasticity strengthens visual activation during SS interpretation, and that in fact crossmodal plasticity is essential to the interpretation ability of SS^2^,^9^,^13^,^19^,^20^. In a few cases, intrinsic crossmodal correspondences are mentioned^13^,^21^, or indirect visualization is argued for sighted subjects^6^. The current dataset supports a diminished role of crossmodal plasticity in that trained participants did not significantly outperform the naive in any of the image sets (*p* < 0.002, with Bonferroni multiple comparisons correction) (Table S3). The similarity in recognition performance between the naive and trained indicates that pre-existing crossmodal connections (*i.e.*, crossmodal mappings) were potentially equally important to SS interpretation as crossmodal plasticity (engendered through training). This set of data could be interpreted along the metamodal or multisensory theory of the brain - “metamodal” meaning that sensory regions process a particular type of information (such as shape) rather than a particular modality^2^,^19^,^22^. Our results show multisensory processing of vOICe information via crossmodal correspondences support the metamodal concept that all sensory information is processed in a multisensory context rather than in separate modalities. These conclusions are not due to a ceiling effect (because in 6 of the image sets, the trained did not perform significantly above chance). Therefore the claim that SS pattern interpretation is primarily based on crossmodal plasticity (at least in sighted participants) should be significantly constrained by the data presented in this study.

A key question is what determines the degree of intuitiveness (*i.e.*, the intrinsic mapping) in the stimulus images. For example, may it be conceivable that image complexity be negatively correlated with participants’ performance? To address this question we correlated image complexity measures with the bimodal matching accuracy. The filters used and results are detailed in the supplementary information. Another way to investigate the reason for vOICe sound intuitiveness is by manipulation of the crossmodal mappings used in the vOICe audio-visual encoding; Expt. 2 will use this approach.

### Expt. 2: Matching Images to a Variety of Audio-to-Visual Device Encodings

#### Introduction

Crossmodal mappings underlie the intuitiveness of vOICe encoding. While this is a logical conclusion from the results in Fig. 3, it is not explicitly proven that crossmodal mappings are the elements that make vOICe understandable to the entirely naive. Further, it is unclear whether the current vOICe encoding scheme (*i.e.*, from height to pitch, from left-right to time) is the optimal encoding scheme. Also, it is unclear which crossmodal mappings are the most critical for intuitiveness. To address these issues, we reversed the direction of each of the primary vOICe encodings or crossmodal mappings and then tested the reversed encodings in comparison to the original vOICe encodings. If the encoding/crossmodal mapping inversion significantly reduces the participants’ accuracy at matching images and sounds, then that crossmodal mapping dimension (but with the original direction) is important to effortlessly interpret vOICe. In other words, we are investigating which engineering encoding parameter combinations make the psychological decoding more intuitive but within the same parameter dimensions as the original (vOICe).

The same bimodal-matching task as Expt. 1 was used for testing alternative vOICe audiovisual mappings. Participants tested all different mappings (including the original vOICe audiovisual mapping) in one session. Four image sets for each crossmodal mapping were tested in one block of trials, with the order of the mapping blocks in random order (the image sets used were chosen for image diversity and significant AV matching performance in Expt. 1). Any degradation in performance due to the confusion of testing many different AV mappings in one session will apply to the original mapping and the modifications, and therefore will be irrelevant to the comparison. Untrained naive participants were tested due to our aim of directly testing the mapping intuitiveness.

#### Results

Results from 8 sighted naive participants indicate that two crossmodal correspondence inversions have a significantly reduced matching accuracy compared to the original encoding (Fig. 4). Matching for reversed brightness and loudness mapping was significantly less accurate for two images sets (*p* < 0.0031, with Bonferroni correction): Interfaces (Welch’s t-test, *p* < 7.28 × 10^−4^) and Natural Textures (Welch’s t-test, *p* < 0.002) (third and fourth image sets in Fig. 4). The reversal of the vertical and horizontal coordinates also degraded performance significantly (*i.e.*, scanning top to bottom and mapping high-low pitches from right to left in reverse encoding; scanning left to right and mapping high-low pitches from top-bottom in original) for one image set: Bars of Different Thickness (Welch’s t-test, *p* < 2.62 × 10^−13^) (first image set in Fig. 4). When all the image sets are summed together, matching for both the brightness and loudness mapping (Welch’s t-test, *p* < 1.60 × 10^−5^) and the orientation of image coordinates (Welch’s t-test, *p* < 4.36 × 10^−8^) when inverted were significantly less accurate than the original vOICe encoding.

#### Discussion

The implication of the crossmodal mapping tests is that two encoding elements are particularly important to image interpretation with vOICe: Brightness correlating with loudness, and the coordinates of the encoding (*i.e.*, scanning from left to right with high pitch at the top as with vOICe, rather than scanning from top to bottom with high pitch at the right).

A reversal or 180 degree rotation of an encoding can apparently be tolerated with these image stimuli (*i.e.*, high-low pitches from bottom to top instead of top to bottom, or scanning right to left instead of left to right). It is likely that this toleration of reversal and 180-degree rotation is due to the left-right and top-bottom symmetry of several of the images used. A 90 degree rotation of the encoding is problematic to interpretation (The “90 degree rotation” means the *x* and *y* axis switch: scanning from top to bottom with high-low pitches from right to left, rather than scanning from left to right with high-low pitches from top to bottom as with vOICe). The problem of switching the *x* and *y* axis encodings further emphasizes the anisotropy of the vOICe encoding (unlike vision), and the importance of displaying information on the *x* axis with vOICe where the highest (temporal) resolution occurs (rather than on the *y* (pitch) axis). In particular, the images that test well with vOICe have information displayed horizontally, and when the encoding axes are switched, the critical information in the *x* direction is less detectable by the lower *y*-axis encoding resolution, thereby reducing accuracy. A comparison of the vertical bar texture naive percent correct (86%) (Fig. 3a) and the horizontal bar texture percent correct (22%) (Fig. 3b) with the vOICe encoding verifies this point.

The brightness-loudness correspondence makes sense, as bright objects in dark areas are the most interesting in natural scenes (rather than vice versa, although with a variety of exceptions). The auditory system is acutely able to recognize the presence of sounds, and is less able to recognize the absence of sounds. These facts suggest that the brightness to loudness translation highlights the most important image elements (*i.e.*, the bright elements), whereas the reverse encoding (darkness translates to loudness) obscures the most important elements. Brown *et al.* studied an exception to this rule, brightness to loudness correlation in auditory SS with dark objects on a light surface. They found the reverse encoding (darkness to loudness) more useful for locating the uncommon dark object^13^.

The testing of vOICe mapping elements showed that crossmodal correspondences underlie vOICe intuitiveness. In particular, naive participants’ ability to match images and vOICe sounds is due in part to two key crossmodal mappings: the brightness to loudness mapping and the orientation of the vOICe mapping. In Expt. 3, we will next investigate if these intuitive vOICe sounds can be processed without attention, or automatically.

### Expt. 3: Matching Images to vOICe Sounds while Performing a Distractor Task

#### Introduction

The naive interpretation of vOICe demonstrated in Expt. 1 above indicates that knowledge of the explicit audiovisual vOICe encoding scheme is not needed for vOICe interpretation. Instead, implicit crossmodal mappings allow the naive interpretation of vOICe sounds. Therefore, the automaticity of the naive interpretation of vOICe depends on the automaticity of the crossmodal correspondences. Crossmodal correspondences (or mappings) can be automatic or not automatic depending on the type of crossmodal mapping and task^23^. One automaticity criterion is attention-load insensitivity, which can be tested by distraction tasks^23^. While the load-insensitivity of crossmodal mappings does not yet appear to have been studied directly, dual task designs have been used to study the impact of high attention load on crossmodal interactions. Alsius and colleagues showed that reduced attention resources limited the McGurk effect^24^. Eramudugolla, *et al.* indicated that the ventriloquist aftereffect can occur under attention load, but is modulated by it^25^. Helbig and Ernst demonstrated that the weighting of visual and haptic stimuli is independent of attention load^26^. These mixed results indicate that some but not all multimodal interactions are load-insensitive.

#### Results

**Figure 2:**
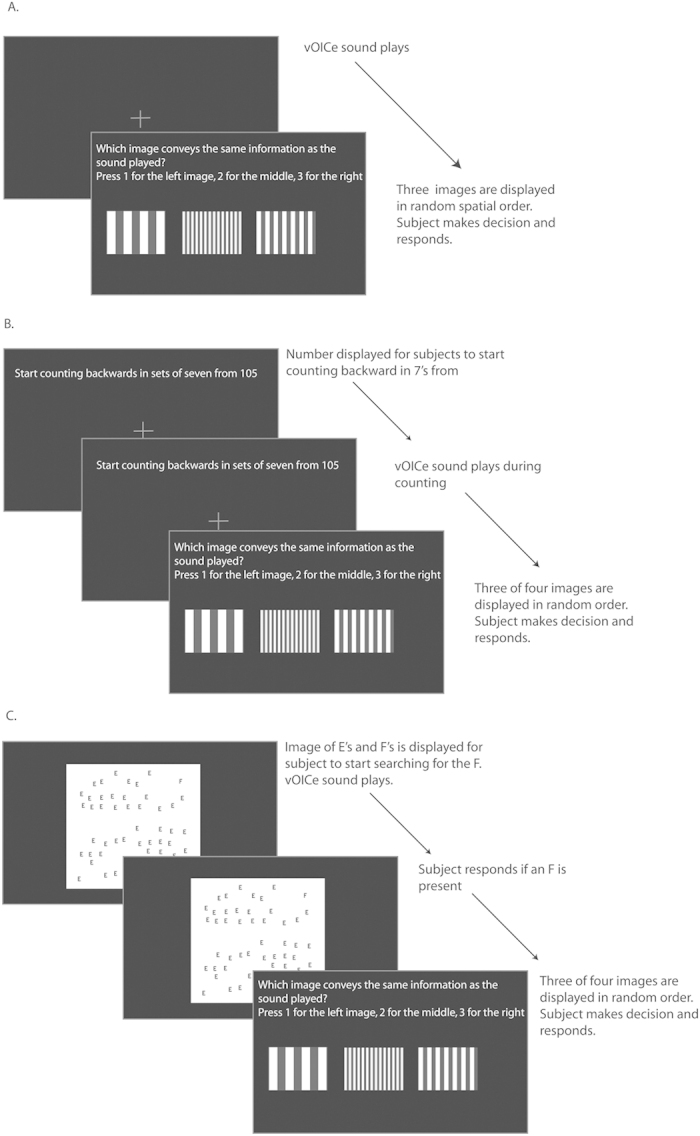
Schematics of procedures for Expts. 1 and 3. (**a**) Is a diagram of the bimodal experiment matching images to sounds with the vOICe encoding (Expt. 1). (**b**) Is a schematic diagram of the audio counting distractor experiment design (Expt. 3). (**c**) Is a schematic diagram of the visual search distractor experiment design (Expt. 3). Schematic and Image generated by N. Stiles.

We tested the load-sensitivity of vOICe interpretation with a distractor task in audition or vision during vOICe sound interpretation (*N* = 8). Participants counted backward silently in sets of 7 from a displayed random number (audio distractor), while the vOICe sound played (Fig. 2b). Participants then matched the vOICe sound to one image of three displayed images that seemed to best match it (3AFC, same design as the bimodal matching experiment with just the counting distractor added). The same participants also performed a visual search distractor task (distraction by serial visual search of the letter F among a set of E’s) of similar design to the counting distractor (Fig. 2c) and the original bimodal experiment (*i.e.*, no distractor) (Note: the original data in Expt. 3 is the same as Expt. 2 original data) (Fig. 2a). Only one of the dual task matching accuracies was significantly different from the original vOICe bimodal matching task for the 4 image sets tested (Fig. 5) (*N* = 8) (*p* < 0.006, with Bonferroni correction) (Natural textures, visual search, *p* < 0.005). When the data are pooled across image sets, both the visual and auditory distractor task accuracies were not significantly different from the original bimodal matching task when a Welch’s t-test is used (*p* < 0.006, with Bonferroni correction) (auditory distractor: *p* < 0.008, visual distractor: *p* < 0.006). When pooled, both distractors are also significantly different than chance (auditory distractor: *p* < 8.36 × 10^−23^, visual distractor: *p* < 4.54 × 10^−21^).

#### Discussion

These results indicate that attention does not play significant role in the processing of vOICe sounds tested but also that vOICe processing can be modulated to some degree by attention.

### Expt. 4: Matching Remembered Labels to vOICe Sounds

Experiment 4 relates the crossmodal matching results to the more conventional, unimodal vOICe training tasks. The vOICe training tasks are mimicked in Experiment 4 with a label memory task (learn and then reiterate a label associated with a given vOICe sound). The performance at the crossmodal matching task and vOICe training (label memory) task were correlated, showing that crossmodal matching used in this paper is related to traditional vOICe training. Details about this experiment are in the supplemental information.

### Expt. 5: Matching Tactile Patterns to vOICe Sounds in the Blind

#### Introduction

Naive sighted participants perform remarkably well matching *visual* images to sounds (Exp. 1–4). But the real question more directly relevant to sensory substitution to aid the blind should be whether naive blind participants can use crossmodal mappings to match *tactile* patterns (in place of images) to vOICe sounds. Thus, we tested both blind and sighted participants on matching sounds to tactile (relief) patterns (Fig. 6a), in a procedure analogous to the original auditory-visual Expt. 1. The relief patterns (in which relief height represented image brightness) corresponded to the visual images described above for lines of different thicknesses and circle patterns of different sizes.

#### Results

Blind participants performed above chance (33%) both before and after training at matching vOICe sounds to the tactile patterns. Following vOICe training, the blind performed significantly above chance for both textures tested (*p* < 0.004, with Bonferroni correction) (Fig. 6b, Bars of different thickness: Blind naive (*N* = 4) 46% and *p* < 0.09, Blind trained (*N* = 4) 65% and *p* < 4.77 × 10^−5^, Sighted naive (*N* = 4) 79% and *p* < 6.30 × 10^−10^; Dots of different sizes: Blind naive (*N* = 4) 44% and *p* < 0.16, Blind trained (*N* = 4) 58% and *p* < 0.001, Sighted naive (*N* = 4) 67% and *p* < 1.41 × 10^−5^). When the subject groups were compared for each image set, only the blind naive and the sighted naive were significantly different from each other for 1 out of 2 of the texture sets (*p* < 0.004, with Bonferroni correction) (Bars of different thickness: *p* < 6.04 × 10^−4^ for Welch’s t-test). When the data are pooled among the texture sets the blind naive and sighted naive are significantly different (*p* < 5.90 × 10^−5^, Welch’s t-test) from each other, respectively.

#### Discussion

Although the blind data for tactile-auditory matching is weaker than the sighted data for auditory-visual matching, it may be in part due to the inclusion of congenitally blind participants. The blind group included two late blind participants and two congenitally blind participants; the late blind performing the better of the two blindness types (average across image sets: Late blind naive (*N* = 2) 50%, Congenitally blind naive (*N* = 2) 40%, Late blind trained (*N* = 2) 71%, Congenitally blind trained (*N* = 2) 52%). The late blind may have performed better because of past visual experiences, which enhanced their spatial perception and increased the pre-existing spatial crossmodal mappings. In addition, the late blind will also likely have a hidden vision-audition intrinsic mapping from past visual experience that does not appear on the tactile-audition matching test performance. Such a hidden visual-auditory mapping may assist in the learning of vOICe by the late blind. However, due the small number of subjects the only definite conclusion from this experiment is that the blind can match tactile patterns to sounds following vOICe training and experience with the matching task (the task was performed by same participants before training).

### General Discussion

Sensory substitution has been assumed to be top-down controlled (particularly in initial stages) and to require extensive attention. This assumption is not always true. Entirely naive participants were found to match vOICe sounds to images significantly above chance (Expt. 1). Therefore, explicit knowledge of the vOICe vision-to-auditory encoding scheme was not required for interpretation of vOICe sounds. In addition, the similarity of the naive and trained performance in these experiments indicates that pre-existing crossmodal interactions (or mappings) may play an equal role in SS pattern recognition as crossmodal plasticity.

The intuitive interpretation of vOICe sounds was shown to be due to intrinsic crossmodal mappings associated with the vOICe encoding scheme by generating significant decrements in matching performance when the vOICe encoding scheme was reversed (Expt. 2). In particular, we investigated engineering encoding parameter combinations that made the psychological decoding more intuitive. It was shown that brightness to loudness mapping and time to horizontal/frequency to vertical mapping are important for naive crossmodal interpretation of vOICe sounds. This result shows that crossmodal mappings are important to vOICe interpretation, while it leaves open the question as to whether attention is needed to interpret vOICe via crossmodal correspondence. To answer this question, a dual task design was used to test the attention load susceptibility of matching vOICe sounds to the correct images (Expt. 3). The use of crossmodal mappings in naive vOICe interpretation was shown to be partially automatic (*i.e.*, relatively insensitive to attentional load). Therefore, the use of crossmodal mappings to interpret vOICe does not always require significant attention resources, suggesting a similarity to visual experience in the sighted. Furthermore, the auditory-visual intrinsic mappings were shown to impact auditory sensory substitution training implying relevance of these results to training protocols (Expt. 4). Finally, the key results were extended to blind participants (the target users of vOICe) with a vOICe sound to tactile pattern matching task (Expt. 5).

**Figure 3:**
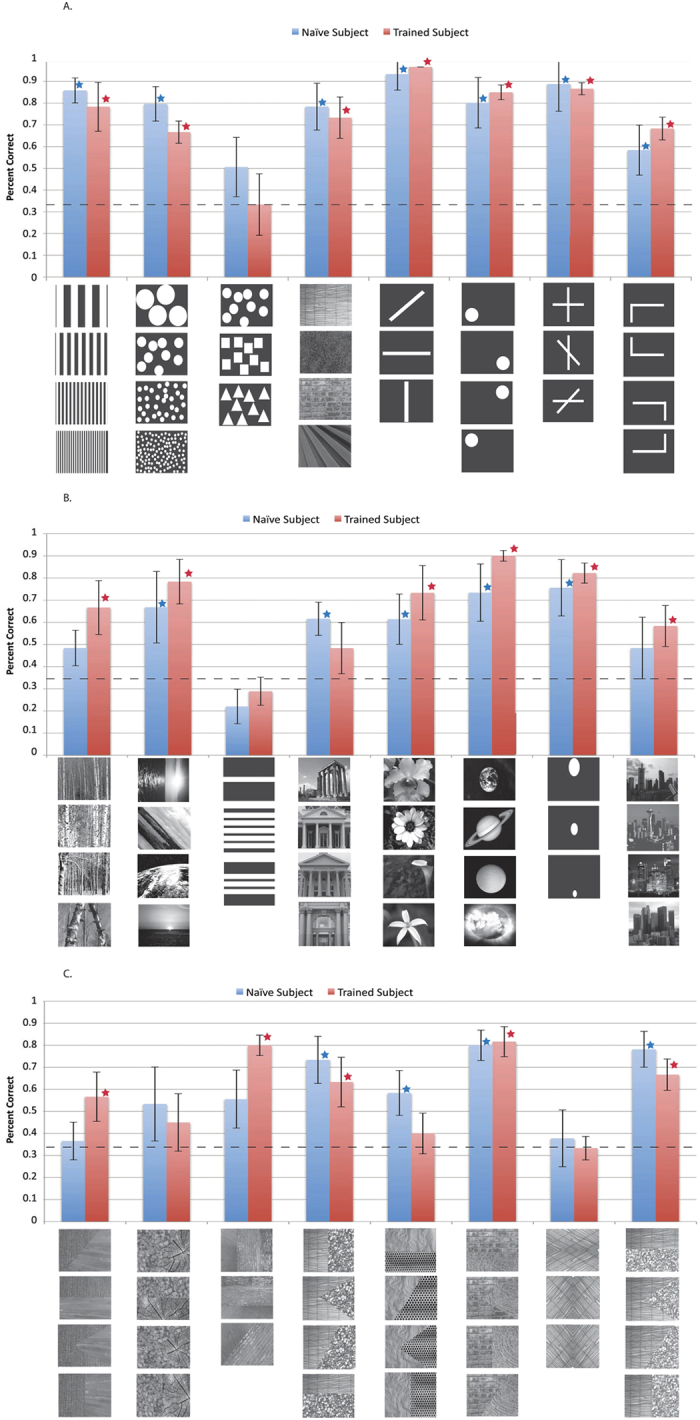
Data from images encoded into vOICe sounds and tested on sighted naive (*N* = 5 to 7) and trained participants (*N* = 5). Audiovisual (AV) matching experiment data for all images used are displayed (images beneath each vertical bar). The dashed line indicates chance and the error bars represent standard deviation. The Blue bars represent the naive data and the red bars represent the trained data. In addition, red stars indicate that the trained subjects performed significantly better than chance (*p* < 0.002, with Bonferroni multiple comparisons correction), and the blue stars indicate that the naive subjects performed significantly better than chance (*p* < 0.002). Image sources^28^,^29^,^30^,^31^,^32^,^33^,^34^,^35^,^36^,^37^,^38^,^39^,^40^,^41^,^42^,^43^,^44^,^45^,^46^,^47^,^48^,^49^,^50^,^51^,^52^,^53^,^54^,^55^ are referenced in the supplemental (Note: many of images shown are images similar to the experimental images, as the original images are restricted by copyright, all original images are also referenced in the supplemental and can be viewed here: http://neuro.caltech.edu/page/research/texture-images/). Note: Photos 23, 26, 28, 32, 48, and 49 courtesy of Morguefile.com, under the general Morguefile license (www.morguefile.com/license)^56^.

**Figure 4:**
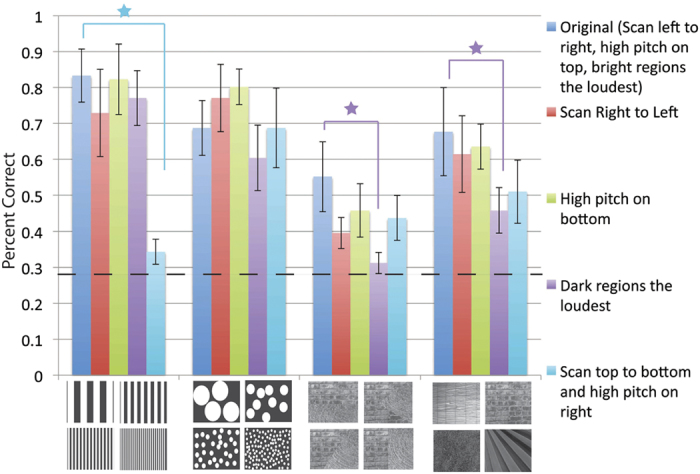
Data and images for matching sounds to images with alternative audio-to-visual (AV) mappings. Naive participants (*N* = 8) matched images and sounds with alternative AV mappings; the original mapping is the vOICe encoding. The error bars represent the standard deviation across participants, and the dashed line represents chance. The stars indicate that the modified mapping is significantly different from the original mapping. Image sources^28^,^29^ are referenced in the supplemental (Note: A few of the images shown are images similar to the experimental images, as the original images are restricted by copyright, all original images are also referenced in the supplemental and can be viewed here: http://neuro.caltech.edu/page/research/texture-images/). Note: Photo 23 courtesy of Morguefile.com, under the general Morguefile license (www.morguefile.com/license).

**Figure 5:**
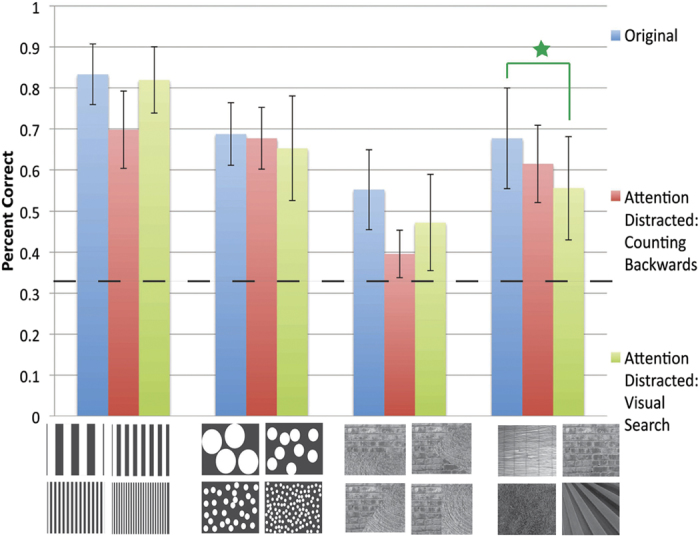
Data and images for matching sounds to images with and without distractors. The results for both visual (*N* = 6) and auditory (*N* = 8) distractor versions in comparison to the original vOICe bimodal matching experiment are plotted in Fig. 5. The error bars represent the standard deviation across participants, and the dashed line represents chance. The star indicates that the distractor version is significantly different from the original mapping. Image sources^28^,^29^ are referenced in the supplemental (Note: A few of images shown are images similar to the experimental images, as the original images are restricted by copyright, so all original images are also referenced in the supplemental and can be viewed here: http://neuro.caltech.edu/page/research/texture-images/). Note: Photo 23 courtesy of Morguefile.com, under the general Morguefile license (www.morguefile.com/license).

Sensory substitution training has been based on the hidden assumption that the primitives of sensory substitution perception will be the same as the primitives of vision, such as dots, lines, and intersections. The visual primitives are often defined as visual elements that are perceptually processed effortlessly and automatically^27^. While sensory substitution is vision-like, it may have crossmodally intuitive primitives that are different from the classical visual primitives. In particular, not all of the visual primitive stimuli were intuitive to interpret with vOICe (*e.g.*, shape texture (naive, 51%), and horizontal bar texture (naive, 22%) (Column 3 of Fig. 3a,b) are not significant). Training protocols that are specially designed to access intrinsic mappings as primitives may enable faster training and greater ease of use. If intuitive stimuli such as textures are the starting point of vOICe training, followed by a gradual increase of image complexity (closer to real-world), participants may learn to use devices more effectively and effortlessly within a shorter training period. For example, pattern recognition of intuitive stimuli could progress to localization of intuitive stimuli on a black background and then to identification and localization of intuitive stimuli within a natural scene. In fact, the identification of subtle texture interfaces has already been tested on naive sighted participants with some success (all of Fig. 3c). This approach differs significantly from the conventional (more effort-demanding) training in which trainees first learn visual geometric primitives and then more natural cluttered scenes constructed from these primitives, with extensive efforts^2^,^10^,^19^.

**Figure 6:**
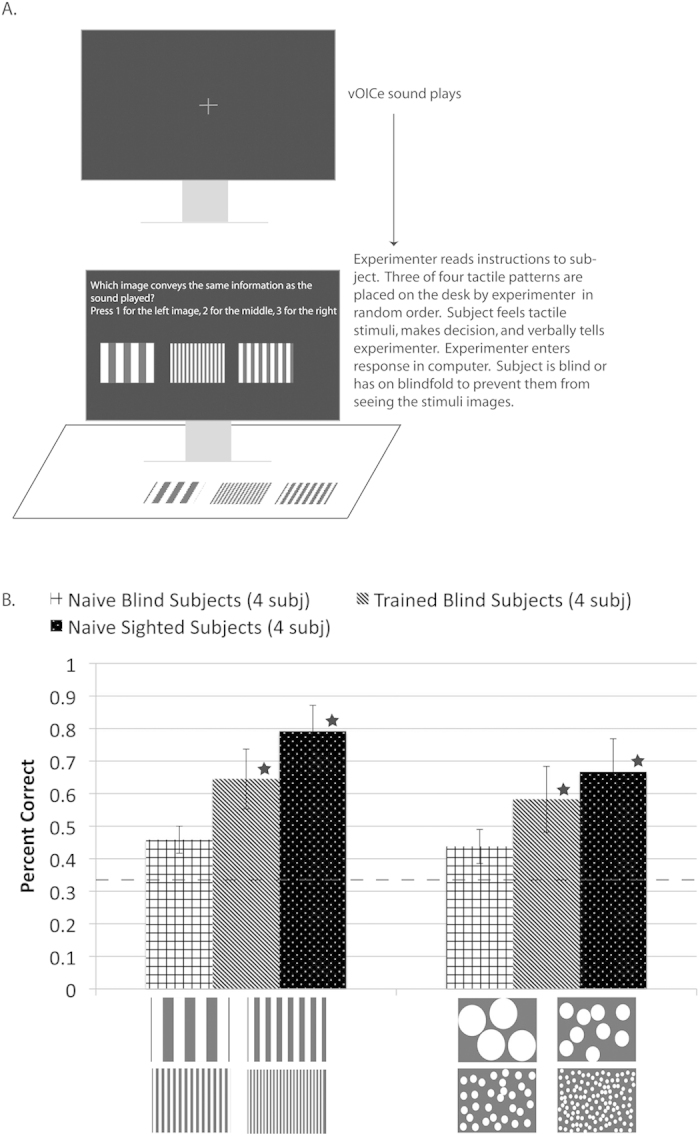
Data and images from matching vOICe sounds and tactile patterns tested on both naive and trained participants. (**a**) Is a diagram of the experiment where blind and sighted participants (sighted participants wore blindfolds) were tested at matching a vOICe sound to one corresponding tactile pattern out of three presented (the white regions of the tactile patterns are raised relative to the black regions). The data for the experiment is presented in (**b**). The grid bars represent naive blind, the gray bars (fine diagonal pattern) represent trained blind, and the black bars (white polka dot) represent naive sighted subjects. The error bars represent the standard deviation across participants and the dashed line represents chance for (**b**). In addition, stars indicate that the subjects performed significantly better than chance (*p* < 0.004, with Bonferroni correction). It is useful to note that the blind subjects were tested naively, then trained and tested again post-training, therefore total subjects is *N* = 4 (this method was required because of the difficulty of recruiting blind participants) (Table S2)). Schematic and Image generated by N. Stiles.

The results of these experiments have profound implications as to how different modalities are intrinsically mapped in the brain, significantly extending previous findings on synaesthesia and intrinsic correspondence. In particular, both texture and natural stimuli employed in the current study demonstrate that crossmodal mapping strength is resistant to the complexity of the encoded images (*i.e.*, even at high image complexity, vOICe sound matching was above chance; Supplementary Fig. S1). A load-sensitivity criterion for automaticity was tested herein on crossmodal mappings for the first time, to the best of our knowledge. Previously, auditory-visual correspondences have shown some evidence of being goal-directed (*i.e.*, not automatic), but to the contrary are speeded up in the Implicit Associations Test (*i.e.*, automatic)^23^,^56^. Expt. 3 used a distraction task to strengthen the case that crossmodal mappings can be automatic with both visual and auditory distractors, indicating partial attentional load independence. Finally, it was shown that crossmodal mappings between audition and tactile perception exist in the blind (Fig. 6b). Despite losing one sense (vision) with the consequence of extensive cortical reorganization, multimodal interactions among the remaining senses stay intact. This result is important to a complete understanding of neural processing in the blind.

While the result that sensory substitution with texture stimuli could be intuitive is exciting, this does not mean that the sensory substitution training is not necessary, nor the question about the optimal training is solved. Rather, this study indicates that some static stimuli (single images and sounds, rather than real-time video-to-sound conversion) when using a forced-choice paradigm (*i.e.* multiple choice) can be intuitive. In order for these results to aid the blind, further research applying these principles to dynamic training paradigms needs to be attempted (as suggested earlier).

Crossmodal mappings enable sensory substitution interpretation, and if used in device training and design could dramatically improve functional outcomes. In particular, the matching of engineered SS audio-visual mappings to intrinsic sensory mappings is important to improving the intuitiveness of sensory substitution. The widespread usage of sensory substitution may be enabled by the use of optimal training paradigms that induce vigorous crossmodal plasticity. Furthermore, even better devices and treatments may be generated by a deeper understanding of sensory integration in the brain via crossmodal mappings.

## Methods

### Experiment 1

The role of crossmodal correspondences was tested with naive (*N* = 5 to 7) and trained sighted (*N* = 5) participants in a bimodal matching task (Expt. 1) (one participant also performed Expt. 4, Table S2). First, all stimuli were presented as a preview (a set of three or four images in random order, 4 seconds each, then a set of three or four vOICe sounds in random order, 1 second each), and then participants heard one sound from the preview and were asked to match one of three presented images to the sound (3AFC) (the preview and test are repeated for each image set). Naive participants were not told the vOICe encoding scheme, nor that the sounds were from the vOICe device. Participants were asked to match the sound to the image that carried the same information; if uncertain, participants were told to guess. Feedback on performance was not provided to participants. Images were compared in sets of three or four so that particular image features and types could be tested separately. Image types ranged from natural to artificial images, and from simple to complex images. Image sets included vertical bar textures of different thicknesses, circular patterns of different element sizes, and images of natural textures (Fig. 3). All images were presented in grayscale, as vOICe sounds do not convey color information. A total of 24 image sets were tested; all images are included in Fig. 2. The naive sighted participants are different than the trained participants. The vOICe frame rate (or refresh rate) was 1 *Hz* for all of the experiments, including Expt. 1, and subject training. A t-test was used to determine significance relative to chance (MATLAB t-test function (ttest), two-tailed test, an alpha of 0.05); all data were tested for normality using the three-sigma rule and passed. Naive and trained data (including the pooled t-test) were compared with a Welch’s t-test (MATLAB ttest2 function, two-tailed test, an alpha of 0.05, unequal variances), where the variances are not assumed to be the same.

We should note that literature studies with task performance improvement with SS, repeat the task in each training session generating task familiarity learning, and use a real-time device generating hand-camera orientation learning. Our task removed the task familiarity learning and hand-camera orientation learning, in order to isolate recognition ability improvement.

The trained sighted participants for Expt. 1 were trained for 8 days, about 1 hour per day on the vOICe device. The vOICe training covered basic object localization and recognition, as well as two constancy tasks (rotation and shape constancy). Training with sensorimotor feedback was used to make the training as close to real world usage as possible within laboratory and safety limits. The training was also used in different constancy experiment, which required many different types of training designs for sensorimotor evaluations of participants and the specific testing protocol. Therefore this constancy training on vOICe, which was detailed and extensive, was also used as a vOICe familiarization protocol for this study. More details are in the supplemental information.

### Experiment 2

The crossmodal mappings underlying vOICe interpretation were tested on naive sighted participants (*N* = 8) (Expt. 2). Participants performed a bimodal matching task of the same design as the original (detailed above) but with different encoding schemes to test the value of different crossmodal mappings. Different encoding paradigms were generated by altering the images input into the vOICe encoding software (for example: the inverted coding of dark regions louder than bright regions was generated by inverting image brightness before processing the image with vOICe software). The encoding inversions tested were: Dark regions louder than bright regions, scanning right to left, high frequency on the bottom, and scanning top to bottom with high frequency on the right (Fig. 1a depicts the original vOICe encoding). The order of testing of the different encoding inversions on participants was randomized. All participants completed all five of the different encoding types (one original and four inversions) in one session. A Welch’s t-test was used to determine significance relative to the original mapping percent correct (MATLAB ttest2 function, two-tailed test, an alpha of 0.05, unequal variances); all data were tested for normality using the three-sigma rule and passed.

### Experiment 3

Automaticity of vOICe interpretation via an attention load experiment was tested with a dual task design (Expt. 3). In the first task of Expt. 3, participants counted backward in sets of 7 from a displayed random number, while listening to the vOICe sound played (vOICe sound started 10 seconds after counting started) (*N* = 8) (same participants as Expt. 2, one participant in common with Expt. 4, Table S2). Participants then matched the vOICe sound to one image of three images displayed (3AFC, same design and image specifications as the bimodal matching experiment). The same participants also performed the original bimodal experiment (*i.e.*, with no counting) in the same session, which was used for comparison (*N* = 8). As with the original experiment, all images and sounds were displayed before the experiment began in random order. A subset of the same participants performed a visual search distraction task in a second session (*N* = 6) (one participant in common with Expt. 4, Table S2). These participants searched for an F within 50 E’s randomly placed in a 100 by 100 location grid within a 1000 × 1000 pixel display field. E and F locations were jittered vertically and horizontally by up to 50 pixels. An F was present in half of the trials and absent in half. The visual search image was 25.4 cm by 25.4 cm, and each letter was 0.6 cm by 1.3 cm on the screen. Participants sat about 63.5 cm from the 27 inch (68.6 cm) iMac screen where the images were presented. The vOICe sound played at the beginning of the visual search task. The participant was instructed to continue searching while the sound was played. Participants responded if an F was present or absent (pressed 1 if present and 2 if absent). Participants then matched the vOICe sound to one image of three images displayed (3AFC, same design and image specifications as the bimodal matching experiment). As with the original experiment, all images and sounds were displayed before the experiment began in random order. A t-test was used to determine significance relative to the original mapping percent correct (Welch’s t-test: MATLAB ttest2 function, two-tailed test, an alpha of 0.05, unequal variances). A t-test was used to determine significance relative to chance (MATLAB t-test function (ttest), two-tailed test, an alpha of 0.05); all data were tested for normality using the three-sigma rule and passed.

### Experiment 4

Sighted naive participants also performed a vOICe memory task (mimicking the vOICe training tasks) (*N* = 8) (Expt. 4) (one participant also performed Expt. 1, another different participant performed Expts 2 and 3, Table S2). Initially, the sounds from vOICe were played in random order twice, and a label (1 through 4) was given to each of the sounds. Then in each trial one of the sounds was played again and the participant responded with the number that matches that sound. This memory task was performed on the same sets of images that were used for the bimodal matching task (only image sets with four images were used in this experiment). A correlation analysis calculated the p-value for Pearson's correlation using a Student's t distribution (MATLAB corr function, two-tailed test); all data were tested for normality using the three-sigma rule and passed.

### Experiment 5

Auditory to tactile mappings were tested via a bimodal matching task (Expt. 5) (naive sighted *N* = 4, naive blind *N* = 4, trained blind *N* = 4 (2 late blind participants and 2 congenitally blind participants)). It is useful to note that the blind subjects were tested naively, then trained and tested again post-training, therefore total number of subjects remained as *N* = 4 (this method was required because of the difficulty of recruiting blind participants) (Table S2)). The setup of the experiment was similar to the visual auditory bimodal matching. As a preview, three to four tactile patterns (10.2 cm by 8.3 cm) were explored and the associated vOICe sounds were played in random order. Participants then listened to one of the vOICe sounds and matched it to one of three tactile patterns presented on a desk surface (3AFC). Participants were asked to match the sound to the tactile pattern that carried the same information. The auditory-tactile matching task instructions were read aloud to the blind or blindfolded sighted, and the participant’s responses (conveyed orally) were recorded by the experimenter. Tactile stimuli were placed in front of the participants on a desk surface for exploration by the participant. Tactile patterns used were generated from black and white images containing two brightness levels by adhering cardstock to the white regions, thereby raising them relative to the black regions. The blind participants were trained for 10 days, 1 hour per day on the vOICe device. The vOICe training covered basic object localization and recognition, as well as on two constancy tasks (rotation and shape constancy). Additional vOICe training information is in the supplemental information. A t-test was used to determine significance relative to chance (MATLAB t-test function (ttest), two-tailed test, an alpha of 0.05); all data were tested for normality using the three-sigma rule and passed. The blind and sighted subject groups were compared with a Welch’s t-test (including the pooled t-test) (MATLAB ttest2 function, two-tailed test, an alpha of 0.05, unequal variances), where the variances are not assumed to be the same.

### General Methods

Additional methods information is provided in the supplemental information. Caltech Internal Review Board approved all experiments in this study. Informed consent was obtained from all study participants and all experiments were performed in accordance with approved guidelines. All t-tests perform the statistical comparison on data pooled across trials.

## Additional Information

**How to cite this article**: Stiles, N. R. B. and Shimojo, S. Auditory Sensory Substitution is Intuitive and Automatic with Texture Stimuli. *Sci. Rep.* **5**, 15628; doi: 10.1038/srep15628 (2015).

## Supplementary Material

Supplementary Information

Supplemental Movie 1

## References

[b1] Meijer P. An experimental system for auditory image representations. Biomedical Engineering, IEEE Transactions on 39, 112–121 (1992). 10.1109/10.121642 1612614

[b2] Amedi A. *et al.* Shape conveyed by visual-to-auditory sensory substitution activates the lateral occipital complex. Nat. Neurosci. 10, 687–689 (2007). 17515898 10.1038/nn1912

[b3] Auvray M., Hanneton S. & Regan O, J. K. Learning to perceive with a visuo-auditory substitution system: Localisation and object recognition with the vOICe. Perception 36, 416 (2007). 17455756 10.1068/p5631

[b4] Bach-y-Rita P., Collins C. C., Saunders F. A., White B. & Scadden L. Vision substitution by tactile image projection. Nature 221, 963–964 (1969). 5818337 10.1038/221963a0

[b5] Bach-y-Rita P., Kaczmarek K. A., Tyler M. E. & Garcia-Lara J. Form perception with a 49-point electrotactile stimulus array on the tongue: A technical note. J. Rehabil. Res. Dev. 35, 427–430 (1998). 10220221

[b6] Poirier C., De Volder A. G. & Scheiber C. What neuroimaging tells us about sensory substitution. Neurosci. Biobehav. Rev. 31, 1064–1070 (2007). 17688948 10.1016/j.neubiorev.2007.05.010

[b7] Proulx M. J., Stoerig P., Ludowig E. & Knoll I. Seeing where through the ears: effects of learning-by-doing and long-term sensory deprivation on localization based on image-to-sound substitution. PloS one 3, 10.1371/journal.pone.0001840 (2008). PMC2267489 18364998

[b8] Chebat D. R., Schneider F. C., Kupers R. & Ptito M. Navigation with a sensory substitution device in congenitally blind individuals. Neuroreport 22, 342 (2011). 21451425 10.1097/WNR.0b013e3283462def

[b9] Merabet L. B. *et al.* Functional recruitment of visual cortex for sound encoded object identification in the blind. Neuroreport 20, 132–138, 10.1097/WNR.0b013e32832104dc (2009). 19104453 PMC3951767

[b10] Collignon O., Lassonde M., Lepore F., Bastien D. & Veraart C. Functional cerebral reorganization for auditory spatial processing and auditory substitution of vision in early blind subjects. Cereb. Cortex 17, 457 (2007). 16581983 10.1093/cercor/bhj162

[b11] Reynolds Z. & Glenney B. in Proceedings of Asia-Pacific Computing and Philosophy. 131–134 (2009).

[b12] Maidenbaum S., Abboud S. & Amedi A. Sensory substitution: Closing the gap between basic research and widespread practical visual rehabilitation. Neurosci. Biobehav. Rev. 41, 3–15 (2014). 24275274 10.1016/j.neubiorev.2013.11.007

[b13] Brown D., Macpherson T. & Ward J. Seeing with sound? Exploring different characteristics of a visual-to-auditory sensory substitution device. Perception 40, 1120–1135 (2011). 22208131 10.1068/p6952

[b14] Spence C. Crossmodal correspondences: A tutorial review. Attention, Perception, & Psychophysics 73, 971–995 (2011). 10.3758/s13414-010-0073-7 21264748

[b15] Stevens J. C. & Marks L. E. Cross-modality matching of brightness and loudness. Proc. Natl. Acad. Sci. USA 54, 407 (1965). 5217429 10.1073/pnas.54.2.407 PMC219679

[b16] Pratt C. C. The spatial character of high and low tones. J. Exp. Psychol. 13, 278 (1930).

[b17] Guzman-Martinez E., Ortega L., Grabowecky M., Mossbridge J. & Suzuki S. Interactive coding of visual spatial frequency and auditory amplitude-modulation rate. Curr. Biol. 22, 383–388 (2012). 22326023 10.1016/j.cub.2012.01.004 PMC3298604

[b18] Ramachandran V. S. & Hubbard E. M. Synaesthesia–A Window into Perception, Thought and Language. Journal of consciousness studies 8, 3–34 (2001).

[b19] Striem-Amit E., Cohen L., Dehaene S. & Amedi A. Reading with sounds: sensory substitution selectively activates the visual word form area in the blind. Neuron 76, 640–652 (2012). 23141074 10.1016/j.neuron.2012.08.026

[b20] Ptito M., Moesgaard S. M., Gjedde A. & Kupers R. Cross-modal plasticity revealed by electrotactile stimulation of the tongue in the congenitally blind. Brain 128, 606–614 (2005). 15634727 10.1093/brain/awh380

[b21] Kim J. K. & Zatorre R. J. Generalized learning of visual-to-auditory substitution in sighted individuals. Brain Res. 1242, 263–275 (2008). 18602373 10.1016/j.brainres.2008.06.038

[b22] Pascual-Leone A. & Hamilton R. The metamodal organization of the brain. Prog. Brain Res. 134, 427–445 (2001). 11702559 10.1016/s0079-6123(01)34028-1

[b23] Spence C. & Deroy O. How automatic are crossmodal correspondences? Conscious. Cogn. 22, 245–260 (2013). 23370382 10.1016/j.concog.2012.12.006

[b24] Alsius A., Navarra J., Campbell R. & Soto-Faraco S. Audiovisual integration of speech falters under high attention demands. Curr. Biol. 15, 839–843 (2005). 15886102 10.1016/j.cub.2005.03.046

[b25] Eramudugolla R., Kamke M. R., Soto-Faraco S. & Mattingley J. B. Perceptual load influences auditory space perception in the ventriloquist aftereffect. Cognition 118, 62–74 (2011). 20979992 10.1016/j.cognition.2010.09.009

[b26] Helbig H. B. & Ernst M. O. Visual-haptic cue weighting is independent of modality-specific attention. Journal of Vision 8, 10.1167/8.1.21 (2008). 18318624

[b27] Treisman A. Preattentive processing in vision. Computer vision, graphics, and image processing 31, 156–177 (1985).

[b28] Patterson A. *Palm Leaf*, http://www.everystockphoto.com/photo.php?imageId=150937&searchId=258289f9cfaa4451809501ad1d1f21d1&npos=63 (2005) Date of access: 05/19/2015.

[b29] SharonAFS. *Bamboo Fencing*, http://www.everystockphoto.com/photo.php?imageId=25399041&searchId=b3a6c3ab50aa0f302f00505bf0896767&npos=151 Date of access: 05/19/2015.

[b30] Superior National Forest. *Birch Stand*, http://www.everystockphoto.com/photo.php?imageId=15945220&searchId=3585247257e557a5d2de29c9f47bfb23&npos=140 Date of access: 05/19/2015.

[b31] Jak. *DSCF1683_t.JPG*, http://www.everystockphoto.com/photo.php?imageId=5028853&searchId=3585247257e557a5d2de29c9f47bfb23&npos=7 (2007) Date of access: 05/19/2015.

[b32] V31S70. *United We Stand.* http://www.everystockphoto.com/photo.php?imageId=19574008&searchId=3585247257e557a5d2de29c9f47bfb23&npos=145 Date of access: 05/19/2015.

[b33] Mnisbett. *113882046411.jpg*, http://www.everystockphoto.com/photo.php?imageId=122017&searchId=3585247257e557a5d2de29c9f47bfb23&npos=23 (2006) Date of access: 05/19/2015.

[b34] T3rmin4t0r. *Into the Sunset*, http://www.everystockphoto.com/photo.php?imageId=2540654&searchId=15dc76442a8bb4c7c7c22ce2ea787d6a&npos=144 Date of access: 05/19/2015.

[b35] Forbes B. *Strathearn*. http://www.everystockphoto.com/photo.php?imageId=5952462&searchId=06664a95d74ecf9fd4e2115e0c7f8147&npos=17 Date of access: 05/19/2015.

[b36] DonkeyHotey. *Earth Horizon*, http://www.everystockphoto.com/photo.php?imageId=14197396&searchId=b93e8669c3d3a2e752ac6bd0ecae8301&npos=84 Date of access: 05/19/2015.

[b37] Siilur.IMG*_5355.JPG*, http://www.everystockphoto.com/photo.php?imageId=138959&searchId=15dc76442a8bb4c7c7c22ce2ea787d6a&npos=107 (2005) Date of access: 05/19/2015.

[b38] Poh K. *The Temple of Olympian Zeus*, http://www.everystockphoto.com/photo.php?imageId=11629832&searchId=ad7fc865073170ee3ada387bf350589b&npos=52 Date of access: 05/19/2015.

[b39] Afroswede. *Uber Church*, http://www.everystockphoto.com/photo.php?imageId=295994&searchId=54ca84a794888fe8d92834787dfa935a&npos=278> Date of access: 05/19/2015.

[b40] Bowen J. *Monticello*, http://www.everystockphoto.com/photo.php?imageId=3335771&searchId=5b3020b13412439106fa42278bf3652d&npos=43 Date of access: 05/19/2015.

[b41] Davidson P. *Vancouver Architecture*, http://www.everystockphoto.com/photo.php?imageId=49199&searchId=54ca84a794888fe8d92834787dfa935a&npos=103 (2005) Date of access: 05/19/2015.

[b42] Beggs. IMG*_3708*, http://www.everystockphoto.com/photo.php?imageId=584013&searchId=93b1453c8ec5a548389c935052536ccb&npos=89 (2005) Date of access: 05/19/2015.

[b43] Quinet. *It's a great life!* http://www.everystockphoto.com/photo.php?imageId=3548286&searchId=df4b892324bbb648f27734b55c206b4b&npos=45 Date of access: 05/19/2015.

[b44] Macrophile. *Red friends*, http://www.everystockphoto.com/photo.php?imageId=284930&searchId=4ae553599f288583dc0698e1a1ef46b5&npos=6 (2005) Date of access: 05/19/2015.

[b45] Beggerly B. J.IMG*_3649*, http://www.everystockphoto.com/photo.php?imageId=30784&searchId=93b1453c8ec5a548389c935052536ccb&npos=36 (2005) Date of access: 05/19/2015.

[b46] NASA. *Earth From Space – Apollo 17*, http://en.wikipedia.org/wiki/File: Apollo_17_Image_Of_Earth_From_Space.jpeg> (1972) Date of access: 05/20/2015.

[b47] NASA, *HINODE_special5*, http://scienceonatable.org/home/hinode_special5/ (2003) Date of access: 05/20/2015.

[b48] NASA. *Saturn*, http://nssdc.gsfc.nasa.gov/photo_gallery/photogallery-saturn.html > (1981) Date of access: 06/10/2015.

[b49] NASA. *Rhea: Full Moon*, http://www.everystockphoto.com/photo.php?imageId=2030462&searchId=8c8abcd1127702c865d84df4c25508c7&npos=26 Date of access: 05/20/2015.

[b50] Jhderojas. *Skyline de Frankfurt*, http://www.everystockphoto.com/photo.php?imageId=4448101&searchId=59a80ac5356106110f0d446252b9717f&npos=14 Date of access: 05/19/2015.

[b51] Wyzik. *Seattle Skyline with Seaplane*, http://www.everystockphoto.com/photo.php?imageId=511671&searchId=ae98bd5090c6b78a267294076187e807&npos=14 (2005) Date of access: 05/19/2015.

[b52] Askins, D. *Dallas skyline*, http://www.everystockphoto.com/photo.php?imageId=10681824&searchId=36ea2edf460642c76108c2442c2876e7&npos=35> Date of access: 05/19/2015.

[b53] Connors, K. DSC*_5801.jpg*, http://www.everystockphoto.com/photo.php?imageId=4984381&searchId=1818147bf417256351ff2f248e1e9454&npos=10> (2006) Date of access: 05/19/2015.

[b54] Siilur. *Tekstuur.jpg*, http://www.everystockphoto.com/photo.php?imageId=5009168&searchId=13ac9e851c7a7176e030652f226b61f9&npos=148> (2007) Date of access: 05/19/2015.

[b55] Hoesly P. *iPhone Background 75 – Sequins*, http://www.everystockphoto.com/photo.php?imageId=9365746&searchId=947a7af4b2d262d3ac2058dd3ca50a82&npos=1 Date of access: 05/19/2015.

[b56] Parise C. V. & Spence C. Audiovisual crossmodal correspondences and sound symbolism: a study using the implicit association test. Exp. Brain Res. 220, 319–333 (2012). 22706551 10.1007/s00221-012-3140-6

