# Supplementary material for: Auditory Sensory Substitution is Intuitive and Automatic with Texture Stimuli
Source: Sci Rep. 2015 Oct 22;5:15628. doi: 10.1038/srep15628 (PMC4615028; doi:10.1038/srep15628)
Supplement: Supplementary Information [file srep15628-s1.pdf]

Title:

Auditory Sensory Substitution is Intuitive and Automatic with Texture Stimuli

Short Title:

Crossmodal Mappings Make Sensory Substitution Easy

Authors:

Noelle R. B. Stiles and Shinsuke Shimojo

Supplementary Information

Methods

Experiment Setup

Participants performed all tasks at a 27 inch (68.6 cm) iMac computer station with Sony noise cancelling headphones (MDR-NC7), inputting responses using a keyboard. Psychophysics Toolbox and MATLAB were used to code the presentation of instructions and stimuli as well as recording responses. Images were presented in black and white on the iMac screen (image size: 10.2 cm by 8.3 cm) approximately 63.5 cm away from the seated participant, and were displayed until the participant responded. Images were encoded into vOICe sounds using vOICe software (seeingwithsound.com), using a 1 Hz scan rate. Screen brightness and audio loudness were set to be comfortable to the participant. Images used were either retrieved from the internet or generated by the experimenter in Adobe Illustrator. Images retrieved from the internet were occasionally modified in Adobe Illustrator or Adobe Photoshop.

vOICe Training structure

The sighted participants were trained for 8 days, 1 hour per day, and the blind participants

were trained for 10 days, 1 hour per day on the vOICe device. Training was performed individually with the same trainer in each session. Training for both the blind and sighted covered basic object localization and recognition, as well as two constancy tasks (rotation and shape constancy). Training with the vOICe device was always performed at a black felt covered table. Each session included the following tasks (in this order): length constancy, orientation constancy, and localization. Data was recorded for each task. These initial training tasks were followed by additional training for the remaining time in the hour. The additional training started with simple object centering and shape identification in the first session, followed by extended length or orientation constancy training in the following sessions. Initial training included: centering in vOICe a white circle on the black-felt-covered board, recognition of simple objects (such as distinguishing a square, triangle, and circle), distinguishing an "L" from a backward L, an upside-down L, and backward and upside-down L (i.e., a 7). Length constancy training involved estimating the lengths of lines at just one orientation angle at a time (such as just 90 degree lines) and the orientation constancy training involved estimating angles with the head at only one tilt.

vOICe Training: The vOICe device

Participants used a vOICe device to learn the constancy tasks. The vOICe device uses a camera embedded in a pair of sunglasses or a webcam attached externally to glasses. Sighted participants were requested to close their eyes during training and evaluation, and wore opaque glasses and/or a mask to block direct visual input. The camera provided a live video feed of the environment, and a small portable computer was used to encode the video into sound in real time. The vOICe software was obtained online at seeingwithsound.com and was used for the video-to-sound encoding.

The training sessions were video recorded for later data analysis. The participants were informed of the recording and consented to it.

vOICe Training: Orientation constancy task

To evaluate orientation constancy, participants were presented with a bar (3 × 30 cm) at 6 different angles (6AFC: 0, 90, 45, −45, 22, or −22 degrees relative to vertical; clockwise rotations correspond to positive angles) with three potential head positions (vertical, tilted left, or tilted right) while using the vOICe device, and then were asked to determine the orientation angle of the bar. The experimenter placed the bar on a black felt covered wall in front of the seated participant and visually estimated each angle position to be presented to the participant. Participants were told to tilt their head left, right, or vertical (no tilt), and were permitted to determine the head tilt angle that they were most comfortable using in each trial (provided that their head was stationary). One head position was requested for each trial. The subject was seated about 81 cm from the bar to be evaluated. The bar angles and head tilt positions (left, right, or vertical) were randomized for each session with 15 total trials per task performance. Participants performed the task once per session. No visual or tactile controls were performed. Feedback was given following each task trial by the experimenter indicating the correct angle of the bar.

vOICe Training: Length constancy task

To evaluate length constancy, participants were presented with 5 lengths of bars (5AFC: 3 cm by either 9, 12, 15, 18, or 21 cm), while the bar was placed in one of four orientations (0, 90, 45, or −45 degrees relative to vertical; clockwise rotations correspond to positive angles). Participants were asked to determine the length of the bar presented independent of the angle that it was presented at. The subject was seated about 81 cm from the bar to be evaluated. Participants first

performed the task with the vOICe device (original task) and then with vision (touch for the blind; control task) in each session. The bar lengths and angles were randomized in order for each session, which included 20 trials for each task performance (original and then control). Feedback was given following each task trial by the experimenter indicating the correct angle and length of the bar.

vOICe Training: Localization

The localization task was performed at the black-felt-covered table. The trainer would place a white circle in one of five locations on a black felt board (the locations were unknown to the participant), and the participant would locate the circle with vOICe, center the circle in the field of view, and then reach for the circle with one finger. The distance between the participant's reach and the circle's center would be measured as a metric of inaccuracy. Feedback was provided to participants by moving their finger from the reached position to the center of the white circle. Thus, the correct direction and location of the circle was provided through tactile and proprioceptive feedback.

Figure 1b Methods

Figure 1b shows plots of amplitude *vs.* time for a set of vOICe generated sounds. The plots of amplitude *vs.* time were generated in MATLAB by importing the .wav file, averaging the amplitudes for each ear, and plotting the amplitudes for the entire duration of the vOICe sound (1 second duration due to 1 *Hz* scan rate).

Supplementary Table 1 Methods

Supplementary Table 1 (Expt. 1) includes edge metrics that were computed by filtering the images with the edge filter (edge function in MATLAB) or corner filter (cornermetric function in MATLAB), averaging all pixels in each image, and then averaging the set of image

results. To demonstrate each edge filter, an example-filtered image is shown in each row of the table (the unfiltered image is in edge detector title row). The number of brightness levels was computed by calculating the quantity of unique brightness values used of 256 within each image, and averaging this value across images for each image set. Repetiveness was calculated by performing a Fourier transform on the image, determining the amplitude of the strongest frequency, and averaging the amplitude across each image set. All filtering and calculations were performed in MATLAB. Figure S2 displays results for the Laplacian of Gaussian edge filter. All correlation analyses calculated the p-value for Pearson's correlation using a Student's t distribution (MATLAB corr function, two-tailed test).

Expt. 1 Image complexity Measures

To examine image complexity we defined complexity by a set of MATLAB edge filter based metrics (Supplementary Table S1). Edge metrics were computed by filtering the images with the edge filter (edge function in MATLAB) or corner filter (cornermetric function in MATLAB), averaging all pixels in each image, and then averaging the set of image results. To demonstrate each edge filter, an example-filtered image is shown in each row of Supplemental Table S1 (the unfiltered image is in edge detector table title row). Four edge filters were tested: Laplacian of Gaussian (filters images with a Laplacian of Gaussian filter, and the looks for zero crossings), Minimum Eigenvalue (minimum eigenvalue method by Shi and Tomasi), Prewitt (indicates edges where the gradient of the image is the maximum), and Canny (calculates the gradient of the image using the derivative of a Gaussian filter and then indicates the local maxima). Further filter details can be found in the MATLAB function details; all filtering used default settings.

Of the four edge filters tested, the best albeit weak correlation between the filter output and naive participants performance was observed for the Laplacian of Gaussian (LOG) edge detector (*rho* = –0.35, $p < 0.09$), and the best correlation for the trained participant performance was observed for the Prewitt edge detector (*rho* = –0.49, $p < 0.02$) (Supplementary Fig. S1 shows the LOG results and Supplementary Table S1 shows all results, and examples of each of the filters). Additional metrics such as the number of brightness levels and spatial repetitiveness were also used to test correlation with bimodal matching performance, but generated weaker results (Supplementary Table S1). The weak negative correlation between complexity and matching percent correct, on one hand, indicates that complexity may make images less intuitive to interpret. Perhaps "complexity" can partially mask the crossmodal correspondences or dilute the crossmodally relevant information with unimodal noise. On the other hand, the weakness of correlation may indicate that something else, such as the strength of the crossmodal intrinsic mapping, may be a strong mitigating factor. More importantly, a linear fit to the data indicated a performance above chance at even the largest complexity values we tested, for both naive and trained participants (LOG edge detector, Supplementary Fig. S1). Even the "complex" stimuli such as natural textures elicited a well-above-chance performance, likely due to the direct selection of strong crossmodal mappings (such as coarse to fine spatial frequencies; images in Fig. 2).

Expt. 4: Matching Remembered Labels to vOICe Sounds.

The bimodal matching experiments described in Expt. 1-3 demonstrate that participants have the ability to crossmodally match vOICe sounds and images. Nevertheless, it is as yet unclear if this crossmodal matching ability affects more conventional, essentially unimodal (*i.e.,*

auditory only) training with the sensory substitution device. In vOICe device training, participants are presented with an object or stimulus, are allowed to explore or listen to it via the device (without vision), and then told a label for an object such as “pencil” or “square”. The participant is then asked if they can identify the objects when presented in random order. Our memory task was designed to be the same as this memory-based label task in vOICe training, but with intuitive sensory substitution stimuli instead of real objects. Sighted participants were given a label (1 through 4) to remember for each vOICe sound, and were then asked to recall the label when one of vOICe sounds was played (sounds were presented in random order). To demonstrate the relationship between this memory task (modeled on vOICe training) and crossmodal matching ability, the memory task was performed with the same stimuli as in the bimodal matching task (Expt. 1 detailed above) by encoding the images with vOICe, and the correlation between the two tasks was calculated. Participant performance on the vOICe memory task (chance: 25%) significantly correlated with performance on the crossmodal audiovisual matching task (chance: 33%) with $rho = 0.68$ ($p = 0.002$) (Supplementary Fig. S2). It is both interesting and surprising that the vOICe sounds corresponding to the images that were crossmodally intuitive were also easier to remember in this memory task. The result indicates that both the memory task and the crossmodal matching task reflect the same intuitiveness/intrinsicness of crossmodal mappings. Therefore, intrinsic crossmodal mappings provide a common basis for sensory substitution training as well as adaptive behavior and scene perception in the real world with the device.

Supplemental Tables and Figures

Table S1

Detector	*rho* Naive	*p* Naive	*rho* Trained	*p* Trained
**Edge Detectors (MATLAB filters); Original Example Image:** 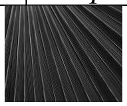				
Laplacian of Gaussian 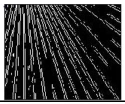	**−0.35**	**0.1**	−0.37	0.08
Minimum Eigenvalue 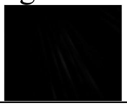	−0.28	0.19	−0.27	0.20
Prewitt 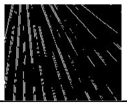	−0.27	0.20	**−0.50**	**0.01**
Canny 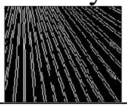	−0.31	0.14	−0.27	0.21
**Other (Original Metrics)**				
Number of Brightness Levels	−0.10	0.65	−0.08	0.70
Repetiveness	−0.28	0.18	−0.50	0.01

Table S1. Results from complexity correlation with bimodal (AV) matching results. Several image filters were used to determine if bimodal matching between vOICe sounds and images correlated with the complexity of the images. This table displays the correlation values, *rho* and *p*, for several different complexity metrics applied to the original images. Edge metrics were computed by filtering the images with the edge filter (edge function in MATLAB) or corner filter (cornermetric function in MATLAB), averaging all pixels in each image, and then averaging the set of image results. To demonstrate each edge filter, an example-filtered image is

shown in each row (the unfiltered image is in edge detector title row). The number of brightness levels was computed by calculating the quantity of unique brightness values used of 256 within each image, and averaging this value across images for each image set. Repetiveness was calculated by performing a Fourier transform on the image, determining the amplitude of the strongest frequency, and averaging the amplitude across each image set. All filtering and calculations were performed in MATLAB. Fig. S1 displays results for the Laplacian of Gaussian edge filter.

Fig. S1.

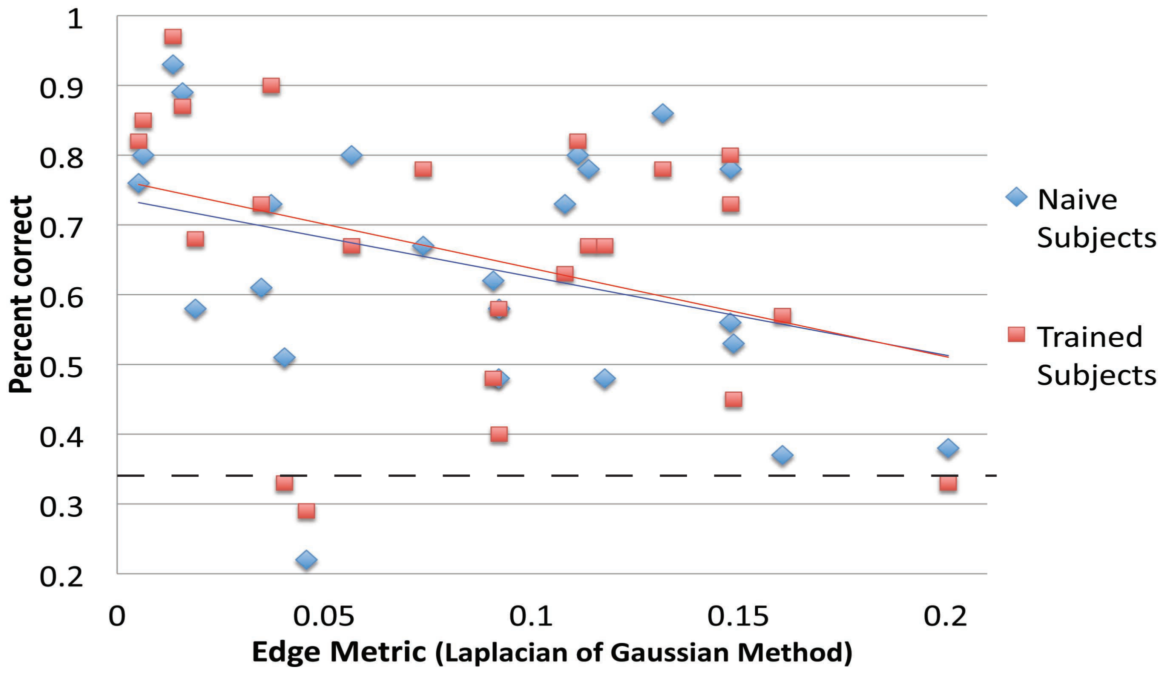

Fig. S1. Correlation between trained and naive bimodal matching data and an edge metric (naive participants: *rho* = –0.35, $p < 0.09$; trained participants: *rho* = –0.39, $p < 0.06$; Chance: 0.33). The complexity quantification was performed in MATLAB. Images were filtered with the Laplacian of Gaussian method (MATLAB edge function) and then spatially averaged to yield a single number per image; the set of numbers was averaged across an image set. The resulting number was correlated with the bimodal audiovisual matching performance. Blue diamonds represent naive subject data, and red rectangles represent trained subject data. The dashed horizontal line indicates chance.

Fig. S2.

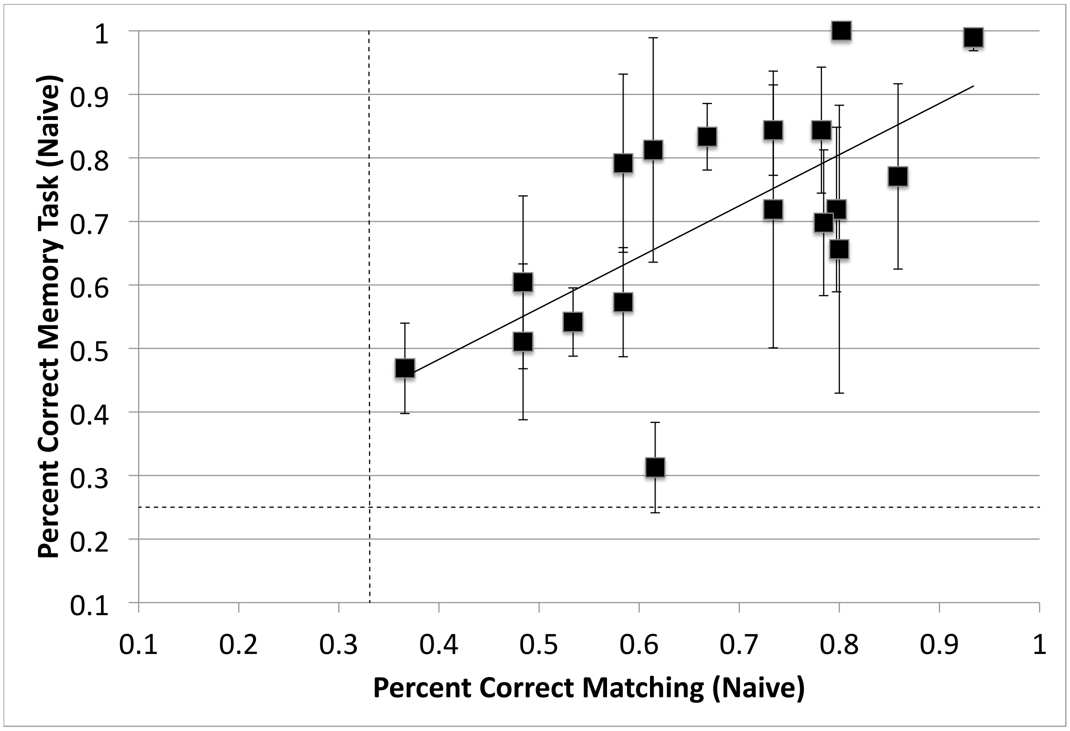

Fig. S2. Correlation between the bimodal matching task (matching vOICe sounds to images) and the unimodal memory task (indicating the remembered label for each vOICe sound). The memory task is the same as most vOICe training tasks. Dashed lines represent chance for both of the tasks and the error bars represent the standard deviation.

Experiment	Expt 1		Expt 2	Expt 3		Expt 4	Expt 5		
Subject Type	Naïve	Trained	Naive	Naive counting	Naive vis search	Naive	Naive sighted	Naive Blind	Trained blind
Initials	1	8	13	13	13	3	27	31	31
	2	9	14	14	14	21	28	32	32
	3	10	15	15	15	22	29	33	33
	4	11	16	16	16	19	30	34	34
	5	12	17	17	17	23			
	6		18	18	19	24			
	7		19	19		25			
			20	20		26			
Subject total	7	5	8	8	6	8	4	4	4

Subjects are identified as numbers to protect identities
Subjects in blue performed more than one experiment (Note: their number is repeated in mutiple Expts)

Table S2. Participant lists for each of the experiments. Each experiment has listed under the type of task the identifying numbers of each of the participants. The numbers in blue are subjects that performed more than one task for the set of experiments in the paper.

Table S3: Experiment 1-5 Data.

Experiment 1 Data

Image Set Name	Trained Subject Avg.	Pval (Trained sig. diff. from chance)	Trained Subject sdev	Naive subject Avg.	Pval (Naive sig. diff. from chance)	Naive subject sdev	Pval (Naive vs. trained sig. diff.) Welch t test
Vertical Stripes	0.783	1.20E-11	0.225	0.857	6.72E-23	0.115	0.2656
Dots Texture	0.667	1.11E-06	0.102	0.798	5.91E-17	0.159	0.0858
Circles, Triangles, or Square Texture	0.333	1	0.283	0.508	0.0078	0.273	0.07
Natural Texture	0.733	3.31E-09	0.190	0.786	5.37E-16	0.216	0.475
Lines of Diff Angles	0.967	5.44E-35	0.000	0.933	3.28E-26	0.148	0.4066
Dots of Diff. Locations	0.850	4.24E-16	0.068	0.800	1.33E-12	0.232	0.4753
Crosses of Diff. Angles	0.867	1.92E-13	0.056	0.889	3.94E-15	0.250	0.7509
L's of Diff. Orientation.	0.683	3.00E-07	0.105	0.583	2.53E-04	0.230	0.2594
Birch Tree Images	0.667	1.11E-06	0.243	0.483	0.0247	0.160	0.0426
Tilted Horizon Images	0.783	1.20E-11	0.201	0.667	1.11E-06	0.323	0.155
Horizontal lines	0.289	0.5188	0.127	0.222	0.0832	0.156	0.4741
Images of Pillars	0.483	0.0247	0.231	0.617	3.53E-05	0.150	0.1445
Images of Flowers	0.733	3.31E-09	0.245	0.617	3.53E-05	0.227	0.1753
Images of Planets	0.900	4.02E-21	0.048	0.733	3.31E-09	0.259	0.0373
Circles of Diff. Sizes	0.822	8.28E-11	0.091	0.756	5.86E-08	0.254	0.4441
Images of City Skylines	0.583	2.53E-04	0.185	0.483	0.0247	0.277	0.2761
Demin/Wood flooring	0.567	6.19E-04	0.224	0.367	0.5972	0.171	0.0282
Leaves/Tree Rings	0.450	0.0768	0.261	0.533	0.0031	0.336	0.3655
Floor/Wall Interface	0.800	9.60E-10	0.093	0.556	0.0049	0.263	0.0129
Bamboo/Circle Pattern	0.633	1.20E-05	0.225	0.733	3.31E-09	0.214	0.2426
Paper/Metal	0.400	0.3002	0.185	0.583	2.53E-04	0.204	0.045
Brick Wall/Large Circular	0.817	1.18E-13	0.136	0.800	1.33E-12	0.137	0.8185
Bamboo Interfaces	0.333	1	0.106	0.378	0.5463	0.258	0.6639
Bamboo/Small Circles	0.667	1.11E-06	0.142	0.783	1.20E-11	0.162	0.155

Experiment 2 Data

Original vOICe Encoding				
	Vertical Stripes	Dots Texture	Interface	Natural Textures
Average	0.8333	0.6875	0.5521	0.6771
Stan. Dev.	0.148	0.153	0.194	0.246
Scan Right to Left				
Average	0.7292	0.7708	0.3958	0.6146
Stan. Dev.	0.243	0.188	0.086	0.213
Pval paired t-test with original	0.0817	0.1958	0.0302	0.3679
High Pitch on Bottom				
Average	0.8229	0.8021	0.4583	0.6354
Stan. Dev.	0.196	0.099	0.148	0.125
Pval paired t-test with original	0.8493	0.0693	0.1958	0.5458
Dark Regions the Loudest				
Average	0.7708	0.6042	0.3125	0.4583
Stan. Dev.	0.153	0.182	0.059	0.126
Pval paired t-test with original	0.2796	0.2295	7.28E-04	0.0021
Scan Top to Bottom and High Pitch on Right				
Average	0.3438	0.6875	0.4375	0.5104
Stan. Dev.	0.070	0.222	0.124	0.175
Pval paired t-test with original	2.62E-13	1	0.1135	0.0186

Experiment 3 Data

Original (8 subjects)

	Vertical Stripes	Dots Texture	Interface	Natural Textures
Average	0.833	0.688	0.552	0.677
Stan. Dev.	0.148	0.153	0.194	0.246

Attention Distracted: Counting Backwards (8 subjects)

	Vertical Stripes	Dots Texture	Interface	Natural Textures
Average	0.698	0.677	0.396	0.615
Stan. Dev.	0.189	0.151	0.116	0.189
Pval Sig. Diff. from Original, Welch's t-test	0.027	0.878	0.030	0.368

Original (6 subjects)

	Vertical Stripes	Dots Texture	Interface	Natural Textures
Average	0.875	0.694	0.583	0.778
Stan. Dev.	0.126	0.180	0.217	0.172

Attention Distracted Visual Search (6 subjects)

	Vertical Stripes	Dots Texture	Interface	Natural Textures
Average	0.819	0.653	0.472	0.556
Stan. Dev.	0.162	0.255	0.234	0.251
Pval Sig. Diff. from Original, Welch's t-test	0.358	0.597	0.184	0.005

Experiment 4 Data

Stimulus sets	vOICe Sounds Avg	vOICe Sounds sdev	Crossmodal Mapping Naive Avg
Vertical Stripes	0.771	0.226	0.859
Dots Texture	0.719	0.263	0.797
Natural Textures	0.698	0.227	0.784
Lines of Diff Angles	0.990	0.029	0.934
Dots of Diff. Locations	1.000	0.000	0.802
L of Diff. Orientations	0.792	0.282	0.584
Birch Tree Images	0.604	0.339	0.484
Tilted Horizon Images	0.833	0.161	0.668
Images of Pillars	0.313	0.222	0.616
Images of Flowers	0.813	0.263	0.614
Images of Planets	0.844	0.163	0.734
Images of City Skylines	0.510	0.246	0.484
Demin/Wood Flooring	0.469	0.248	0.366
Leaves/Tree Rings	0.542	0.199	0.534
Bamboo/Circle Pattern	0.719	0.339	0.734
Paper/Metal	0.573	0.206	0.584
Brick wall/Large Circular	0.656	0.355	0.800
Bamboo/Small Circles	0.844	0.181	0.782

Experiment 5 Data

	Blind Naïve Subjects Avg	Blind Naïve Subjects sdev	Pval (sig. diff. from chance)	Pval (Blind Naive vs. blind trained sig. diff.)
Vertical Stripes	0.458	0.083	0.092	0.066
Dots Texture	0.438	0.105	0.1566	0.156

	Blind Trained Subjects Avg	Blind Trained Subjects sdev	Pval (sig. diff. from chance)	Pval (Blind trained vs. sighted naive sig. diff.) Welch t test
Vertical Stripes	0.646	0.184	4.77E-05	0.115
Dots Texture	0.583	0.203	0.0011	0.404

	Sighted Naive Avg	Sighted Naive sdev	Pval (sig. diff. from chance)	Pval (Blind naive vs. sighted naive sig. diff.) Welch t test
Vertical Stripes	0.792	0.160	6.30E-10	6.037E-04
Dots Texture	0.667	0.204	1.41E-05	0.024

Table S3. Experiment 1-5 Data. Table S3 includes the data from all experiments in the paper including the percent correct (*i.e.* avg., short for average), standard deviations (*i.e.* sdev. or stan. dev.), and the p-values from MATLAB t-tests or Kruskal-Wallis tests (*i.e.* pval) for each of the participant groups (sig. diff is short for significantly different). Note: If the statistical test is not listed, a MATLAB t-test was used.

Movie 1

Movie 1. vOICe texture sound and image demonstration. This video plays the vOICe sounds for two sets of textures, and displays the corresponding images simultaneously.

Replacement Figure References

This section references the images that are shown in the figures in the paper (for the links to the images used in the experiment see the section: "Original Figure References"). Images in Figure 2, 3, and 4 were either generated by N. Stiles or obtained online and modified. The images in Figure 3 and Figure 4 are the same as Figure 2a column 1, 2, and 4, and Figure 2c column 6.

In Figure 2a, image column 1, 2, 3, 5, 6, 7, and 8 were generated by N. Stiles. The images in column 4 partially obtained online from the following sources and the modified by N. Stiles: palm leaf (http://www.everystockphoto.com/photo.php?imageId=150937&searchId=258289f9cfaa4451809501ad1d1f21d1&npos=63), the bamboo (http://www.everystockphoto.com/photo.php?imageId=25399041&searchId=b3a6c3ab50aa0f302f00505bf0896767&npos=151), grass (generated by N. Stiles) and brick wall (generated by N. Stiles).

In Figure 2b, image column 3, and 7 were generated by N. Stiles. The images in column 1 were obtained online from the following sources and then modified by N. Stiles (images in descending order): birch 1 (http://www.everystockphoto.com/photo.php?imageId=15945220&searchId=3585247257e557a5d2de29c9f47bfb23&npos=140), birch 2 (http://www.everystockphoto.com/photo.php?imageId=5028853&searchId=3585247257e557a5d2de29c9f47bfb23&npos=7), birch 3 (http://www.everystockphoto.com/photo.php?imageId=19574008&searchId=3585247257e557a5d2de29c9f47bfb23&npos=145), and birch 4

(http://www.everystockphoto.com/photo.php?imageId=122017&searchId=3585247257e557a5d2de29c9f47bfb23&npos=23). The images in column 2 were obtained online from the following sources and then modified by N. Stiles (descending order): horizon 1 (http://www.everystockphoto.com/photo.php?imageId=2540654&searchId=15dc76442a8bb4c7c7c22ce2ea787d6a&npos=144), horizon 2 (http://www.everystockphoto.com/photo.php?imageId=5952462&searchId=06664a95d74ecf9fd4e2115e0c7f8147&npos=17), horizon 3 (http://www.everystockphoto.com/photo.php?imageId=14197396&searchId=b93e8669c3d3a2e752ac6bd0ecae8301&npos=84), and horizon 4 (http://www.everystockphoto.com/photo.php?imageId=138959&searchId=15dc76442a8bb4c7c7c22ce2ea787d6a&npos=107). The images in column 4 were obtained online from the following sources and then modified by N. Stiles: pillars 1 (http://www.everystockphoto.com/photo.php?imageId=11629832&searchId=ad7fc865073170ee3ada387bf350589b&npos=52), pillars 2 (http://www.everystockphoto.com/photo.php?imageId=3335771&searchId=5b3020b13412439106fa42278bf3652d&npos=43), pillars 3 (http://www.everystockphoto.com/photo.php?imageId=295994&searchId=54ca84a794888fe8d92834787dfa935a&npos=278), and pillars 4 (http://www.everystockphoto.com/photo.php?imageId=49199&searchId=54ca84a794888fe8d92834787dfa935a&npos=103). The images in column 5 were obtained online from the following sources and then modified by N. Stiles: flowers 1 (http://www.everystockphoto.com/photo.php?imageId=584013&searchId=93b1453c8ec5a548389c935052536ccb&npos=89), flowers 2

(http://www.everystockphoto.com/photo.php?imageId=3548286&searchId=df4b892324bbb648f27734b55c206b4b&npos=45), flowers 3 (http://www.everystockphoto.com/photo.php?imageId=284930&searchId=4ae553599f288583dc0698e1a1ef46b5&npos=6), and flowers 4 (http://www.everystockphoto.com/photo.php?imageId=30784&searchId=93b1453c8ec5a548389c935052536ccb&npos=36). The images in column 6 were obtained online from the following sources and then modified by N. Stiles: the earth (http://en.wikipedia.org/wiki/File:Apollo_17_Image_Of_Earth_From_Space.jpeg), the sun (http://scienceonatable.org/home/hinode_special5/), Saturn (http://www.planetsforkids.org/planet-saturn.html), and the moon (http://www.everystockphoto.com/photo.php?imageId=2030462&searchId=8c8abcd1127702c865d84df4c25508c7&npos=26). The images in column 8 were obtained online from the following sources and then modified by N. Stiles: skylines 1 (http://www.everystockphoto.com/photo.php?imageId=4448101&searchId=59a80ac5356106110f0d446252b9717f&npos=14), skylines 2 (http://www.everystockphoto.com/photo.php?imageId=511671&searchId=ae98bd5090c6b78a267294076187e807&npos=14), skylines 3 (http://www.everystockphoto.com/photo.php?imageId=10681824&searchId=36ea2edf460642c76108c2442c2876e7&npos=35), and skylines 4 (http://www.everystockphoto.com/photo.php?imageId=4984381&searchId=1818147bf417256351ff2f248e1e9454&npos=10).

In Figure 2c, image column 1, 3, 5, and 6 were generated by N. Stiles. The images in column 2 were partially obtained online from the following sources and then modified by N. Stiles: leaf

texture (generated by N. Stiles) and wood texture (http://www.everystockphoto.com/photo.php?imageId=5009168&searchId=13ac9e851c7a7176e030652f226b61f9&npos=148). The images in column 4 and 8 were obtained online from the following sources and then modified by N. Stiles: bamboo texture (http://www.everystockphoto.com/photo.php?imageId=25399041&searchId=b3a6c3ab50aa0f302f00505bf0896767&npos=151), dot texture (http://www.everystockphoto.com/photo.php?imageId=9365746&searchId=947a7af4b2d262d3ac2058dd3ca50a82&npos=1). The images in column 7 were obtained online from the following sources and then modified by N. Stiles: bamboo texture (http://www.everystockphoto.com/photo.php?imageId=25399041&searchId=b3a6c3ab50aa0f302f00505bf0896767&npos=151).

Original Figure References

This section references the images used in the experiments (several cannot be shown in the paper due to copyright restrictions, but can be viewed here: http://neuro.caltech.edu/page/research/texture-images/). Images in Figure 2, 3, and 4 were either generated by N. Stiles or obtained online and modified. The images in Figure 3 and Figure 4 are the same as Figure 2a column 1, 2, and 4, and Figure 2c .column 6.

In Figure 2a, image column 1, 2, 3, 5, 6, 7, and 8 were generated by N. Stiles. The images in column 4 were obtained online from the following sources and the modified by N. Stiles: grass (http://tank-battle 3d.googlecode.com/svn/trunk/TankBattle3d/Content/Textures/), brick wall (http://cfrevoir.deviantart.com/art/Seamless-Brick-Wall-Texture-94579094), palm leaf

(http://decibel.fi.muni.cz/models/cinema2012/xgoljer1/2/tex/), and the bamboo (http://bgfons.com/download/1603).

In Figure 2b, image column 3, and 7 were generated by N. Stiles. The images in column 1 were obtained online from the following sources and then modified by N. Stiles (images in descending order): birch 1 (http://photography.national geographic.com/wallpaper/photography/photos/patterns-landscapes/white-birch-trees/), birch 2 (http://www.art.com/products/p 14681429-sa-i3117042/mark-newman-birch-trees-usa.htm), birch 3 (http://elementsofcharlotte.com/blog/ identifying-fall-leaves-in-charlotte/), and birch 4 (http://withlightsteam.com/veniki/). The images in column 2 were obtained online from the following sources and then modified by N. Stiles (descending order): horizon 1 (http://galleryhip.com/sunset-horizon-line.html), horizon 2 and 3 are no longer available online, horizon 4 (http://dict.space.4goo.net/dict?q=horizon). The images in column 4 were obtained online from the following sources and then modified by N. Stiles: pillars 1 (http://greece.greekreporter.com/2013/05/01/international-scientific-conference-in-ancient-olympia/), pillars 2 (https://www.pinterest.com/pin/277252920779537265/), pillars 3 (no longer online), pillars 4 (http://quoteko.com/porch-posts-post-columns-first-class-building-products.html). The images in column 5 were obtained online from the following sources and then modified by N. Stiles: flowers 1 (https://www.flickr.com/photos/93521786@N00/1778211912/), flowers 2 (https://swittersb.wordpress.com/2011/05/07/strawberry-rhubarb-cobbler/simple-daisy-white-flower/), flowers 3 (http://www.giardinaggio.it/linguaggiodeifiori/singolifiori/calla.asp), flowers 4 (beautifulflowerpictures.com/whatsnew.html). The images in column 6 were obtained online from the following sources and then modified by N. Stiles: the Earth

(http://en.wikipedia.org/wiki/File: Apollo_17_Image_Of_Earth_From_Space.jpeg), the sun (http://scienceonatable.org /home/hinode_special5/), Saturn (http://www.planetsforkids.org/planet-saturn.html), and the moon (http://cowbird.com/story/24486/The_Moon_Shines_Bright/). The images in column 8 were obtained online from the following sources and then modified by N. Stiles: skylines 1 (http://un-essai.over-blog.com/categorie-1258796.html), skylines 2 (http://www.wallpapersam.com/wallpaper/seattle-skyline-night-washington.html), skylines 3 (http://www.texascma.org/Conferences/2008/2008_conference.htm), skylines 4 (http://www.pic2fly.com/Fact+About+La+Los+Angeles.html).

In Figure 2c, all images were obtained online and then modified to generate texture interfaces by N. Stiles. The images in column 1 were obtained online from the following sources and then modified by N. Stiles: jean texture (http://fashionforeverr.blogspot.com/2012/01/elements-of-design-texture.html), wood flooring texture (http://www.a-v-designs.com/wood-fireplace/). The images in column 2 were obtained online from the following sources and then modified by N. Stiles: leaf texture (https://www.pinterest.com/pin/308004061986536061/) and wood texture (http://sugarchalet.ca/?attachment_id=131). The images in column 3 were obtained online from the following sources and then modified by N. Stiles: Image (no longer online). The images in column 4 and 8 were obtained online from the following sources and then modified by N. Stiles: bamboo texture (http://bgfons.com/download/1603), dot texture (https://www.pinterest.com/pin/98445941824977011/). The images in column 5 were obtained online from the following sources and then modified by N. Stiles: paper texture (http://junior3d.ru/texture/starayaBumaga.html), metal mesh texture (no longer online). The

images in column 6 were obtained online from the following sources and then modified by N. Stiles: brick texture (http://cfrevoir.deviantart.com/art/ Seamless-Brick-Wall-Texture-94579094), and the circle texture (http://s1120.photobucket.com/user/AndrewRGyr/media/Ceiling%20Textures/Gold_Bead_Halo_). The images in column 7 were obtained online from the following sources and then modified by N. Stiles: bamboo texture (http://bgfons.com/download/1603).
